# Time series flood mapping using the Copernicus dataset in Google Earth Engine of the Mountainous Region

**DOI:** 10.1016/j.dib.2025.112010

**Published:** 2025-08-26

**Authors:** Narayan Thapa, Sujan Nepali, Raman Shrestha, Suman Sanjel

**Affiliations:** a International Center for Integrated Mountain Development, Nepal; b Nepal Open University, Nepal

**Keywords:** Sentinel-2, NDVI Thresholding, Cloud Filling, Cloud Masking, Remote Sensing

## Abstract

In mountainous countries like Nepal, floods are a major challenge due to complex topography, intense snowmelt, and highly variable monsoon rainfall that drive frequent flooding events. This study focuses on the Hilly and Himalayan regions of Nepal, where flood monitoring and risk management are increasingly important for safeguarding vulnerable communities and infrastructure.

This study presents a high-resolution, time-series flood extent dataset derived from the Copernicus Sentinel-2 Level-2A imagery at a 10-meter spatial resolution, covering the years 2019 to 2023. Flood mapping was performed using the Normalized Difference Vegetation Index (NDVI) combined with region-specific thresholding. NDVI values below 0 represent open water, while values between 0 and 0.1 often indicate mud, bare soil. A threshold of NDVI <0.019 was applied to identify flood-affected areas in the hilly region to capture the debris flow type flood, whereas NDVI <0 was used for the Himalayan region, because of the presence of snow and water that complicated classification due to their spectral similarity with other features. Snow-covered areas were masked using the Copernicus Global Land Cover dataset to improve accuracy in the high altitude zones.

Data processing was performed on the Google Earth Engine (GEE) platform. Monsoon-season image composites were generated after applying cloud masking using the Scene Classification Layer (SCL), and temporal cloud gaps were filled using post-monsoon imagery to ensure continuous temporal data. The resulting flood extent maps reveal consistent spatial patterns and provide critical data for flood forecasting, risk-sensitive land use planning, and interdisciplinary studies. Despite challenges with cloud interference and complex terrain, this dataset offers valuable insights into flood dynamics across Nepal’s mountainous landscape.

Specifications Table

**Table:** 

Subject	Earth & Environmental Sciences
Specific subject area	Development of flood extent datasets for the mountainous regions of Nepal using remote sensing.
Type of data	TIFF, ESRI shapefile, CSV
Data collection	The Copernicus Sentinel-2 Level-2A imageries were processed in the free and open-source platform, Google Earth Engine. A cloud-free monsoon composite was generated using the cloud-masking and cloud-filling approach. The Normalized Difference Vegetation Index (NDVI) was calculated, and a region-specific threshold was applied to develop time series flood inventory. The snow-covered regions were masked using the Copernicus Global Land Cover dataset. Administrative boundaries were obtained from the National Geoportal, Survey Department of Nepal, while river networks were sourced from HydroSHEDS.
Data source location	Hilly and Himalayan regions of Nepal
Data accessibility	The dataset is publicly available in the Zenodo repository: Version1: https://doi.org/10.5281/zenodo.15242528 Version2 snow masked: https://doi.org/10.5281/zenodo.15702358
Related research article	None

## 1 Value of the Data

• River monitoring: This dataset provides annual flood extent layers from 2019 to 2023 across the Hilly and Himalayan regions of Nepal, which are essential for early warning systems, flood risk assessment, and disaster management in mountainous terrains.
• Risk-sensitive land use planning: The detailed flood extent maps offer critical spatial information to support risk-sensitive land use planning in mountainous terrain. By identifying flood-prone zones at high spatial resolution, planners can avoid or restrict development in vulnerable areas, minimizing flood damage to infrastructure and communities.
• Scientific research and innovation: This dataset is a valuable resource for scientific research in the fields of hydrology, environmental science, and climate change. Researchers can use the data to study the impacts of floods, develop new models, and create innovative solutions for flood management.
• Machine learning and AI applications: The Researcher can use the dataset to train and validate the machine learning models for susceptibility mapping and predicting future events. The dataset provides a foundation for developing data-driven flood early warning systems and advanced risk assessment models.
• Policy development: Policymakers can use this dataset for informed decision-making, including risk-sensitive land use planning, infrastructure development, and the design of flood-resilient policies.
• Interdisciplinary research: The dataset is relevant across disciplines, including urban planning, public health, and social sciences. It can support studies on flood resilience, social vulnerability, and public health impacts such as waterborne disease outbreaks.

## 2 Background

Flood management in Nepal has evolved significantly since the introduction of the Natural Calamity Relief Act 1982 CE, which primarily focused on the post-flood response [1] due to a lack of a historical dataset. In recent years, more proactive measures have been implemented, including capacity building and resource development in remote sensing technologies, vulnerability mapping, and community-based early warning systems across various regions in Nepal [[2], [3], [4]]. These efforts have been supported by the Disaster Risk Reduction and Management Act 2019, which emphasizes understanding the risk, preparedness, and mitigation approaches [5].

The past research was largely focused on specific regions and isolated case studies [[6], [7], [8]]. There remains a significant gap in time-series flood databases covering the mountainous regions. These areas, characterized by complex landscapes, unpredictable precipitation patterns, and intense snowmelt, experience more frequent flash floods. This study addresses the critical data gap by generating a detailed, time-series flood extent database for the mountainous regions of Nepal using freely available Copernicus datasets, which supports future research, early warning systems, and informed decision-making in flood risk management.

## 3 Data Description

The dataset includes annual flood extent maps for the Hilly and Himalayan regions of Nepal between 2019 and 2023. The dataset is provided in multiple formats: raster (TIFF), vector (shapefile), and tabular Excel (xlsx) formats. Flood mapping was conducted using Sentinel-2 surface reflectance imagery, focusing on the monsoon period (June- October). To address cloud contamination, cloud-masked composites were generated, and additional post-monsoon images (November- February) were used to fill cloud-masked areas. Snow-covered pixels were excluded to reduce misclassification in high altitude zones. The two versions of the datasets are hosted in Zenodo:

• Version 1: https://doi.org/10.5281/zenodo.15242528
• Version 2 (snow-masked): https://doi.org/10.5281/zenodo.15702358

**Repository Structure version 1**

**Area Information XLSX File:**

File Name: AreaHHFlood2019to2023Regionwise.xlsx

Description: This file contains region-wise (hilly and Himalayan regions of Nepal) flood-affected area (in square kilometres) from 2019 to 2023.

**Yearly Flooded TIFF Files:**

File Naming Convention:

**Table:** 

S No.	Name	Description	Pixel value
1	HHFlood19.tif	Hilly Himalayan Region Flood 2019 tiff file	1 represents flood, and 0 represents non-flood
2	HHFlood20.tif	Hilly Himalayan Region Flood 2020 tiff file	
3	HHFlood21.tif	Hilly Himalayan Region Flood 2021 tiff file	
4	HHFlood22.tif	Hilly Himalayan Region Flood 2022 tiff file	
5	HHFlood23.tif	Hilly Himalayan Region Flood 2023 tiff file	

Description: These files represent the yearly flooded areas.

Resolution: 10 m

**Geometry Region File:**

File Name: Hilly_Himalaya_Region.shp

Description: This shapefile defines the study area and contains attribute information for the regions, specifically indicating whether the region is hilly or Himalayan.

**Yearly Flooded vector files:**

File Naming Convention:

**Table:** 

S No.	Name	Description
1	mergedVector_HH_Flood_Erase2019.shp	Hilly Himalayan Region Flood 2019 in vector file
2	mergedVector_HH_Flood_Erase2020.shp	Hilly Himalayan Region Flood 2020 in vector file
3	mergedVector_HH_Flood_Erase2021.shp	Hilly Himalayan Region Flood 2021 in vector file
4	mergedVector_HH_Flood_Erase2022.shp	Hilly Himalayan Region Flood 2022 in vector file
5	mergedVector_HH_Flood_Erase2023.shp	Hilly Himalayan Region Flood 2023 in vector file

Description: These files represent the yearly flooded areas.

**Geometry River files**

File Name: RiverNetworkHillyHimalayan_GCSWGS84.shp

Description: This shapefile defines the river network of Nepal.

**Coordinate System:**

EPSG Code: 32,465

Projection: EPSG:32,465 (WGS 1984 UTM Zone 45 N).

**Yearly Flooded TIFF Files with snow area masked version 2:**

File Naming Convention:

**Table:** 

S No.	Name	Pixels value	Description
1	Flood2019.tif	1 represents flooded pixels	Hilly Himalayan Region Flood 2019 tiff file
2	Flood2020.tif		Hilly Himalayan Region Flood 2020 tiff file
3	Flood2021.tif		Hilly Himalayan Region Flood 2021 tiff file
4	Flood2022.tif		Hilly Himalayan Region Flood 2022 tiff file
5	Flood2023.tif		Hilly Himalayan Region Flood 2023 tiff file

## 4 Experimental Design, Materials and Methods

### 4.1 Methodology

**Fig. 1:**
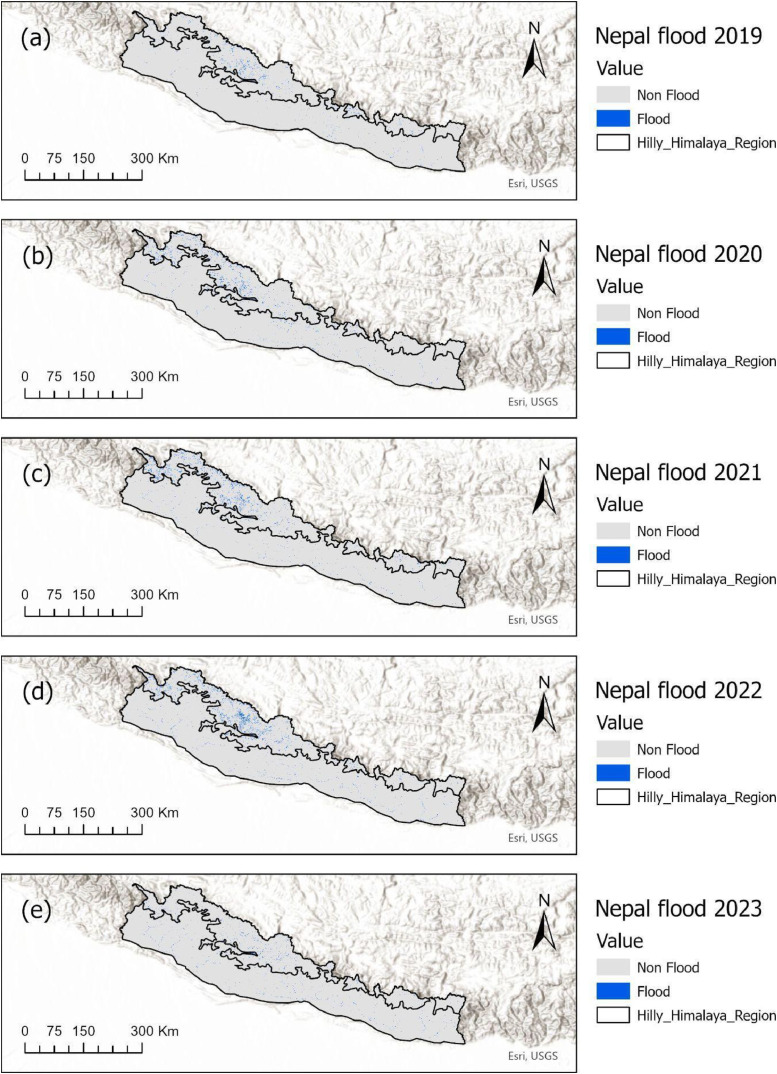
Flood time series data of the Himalayas.

**Fig. 2:**
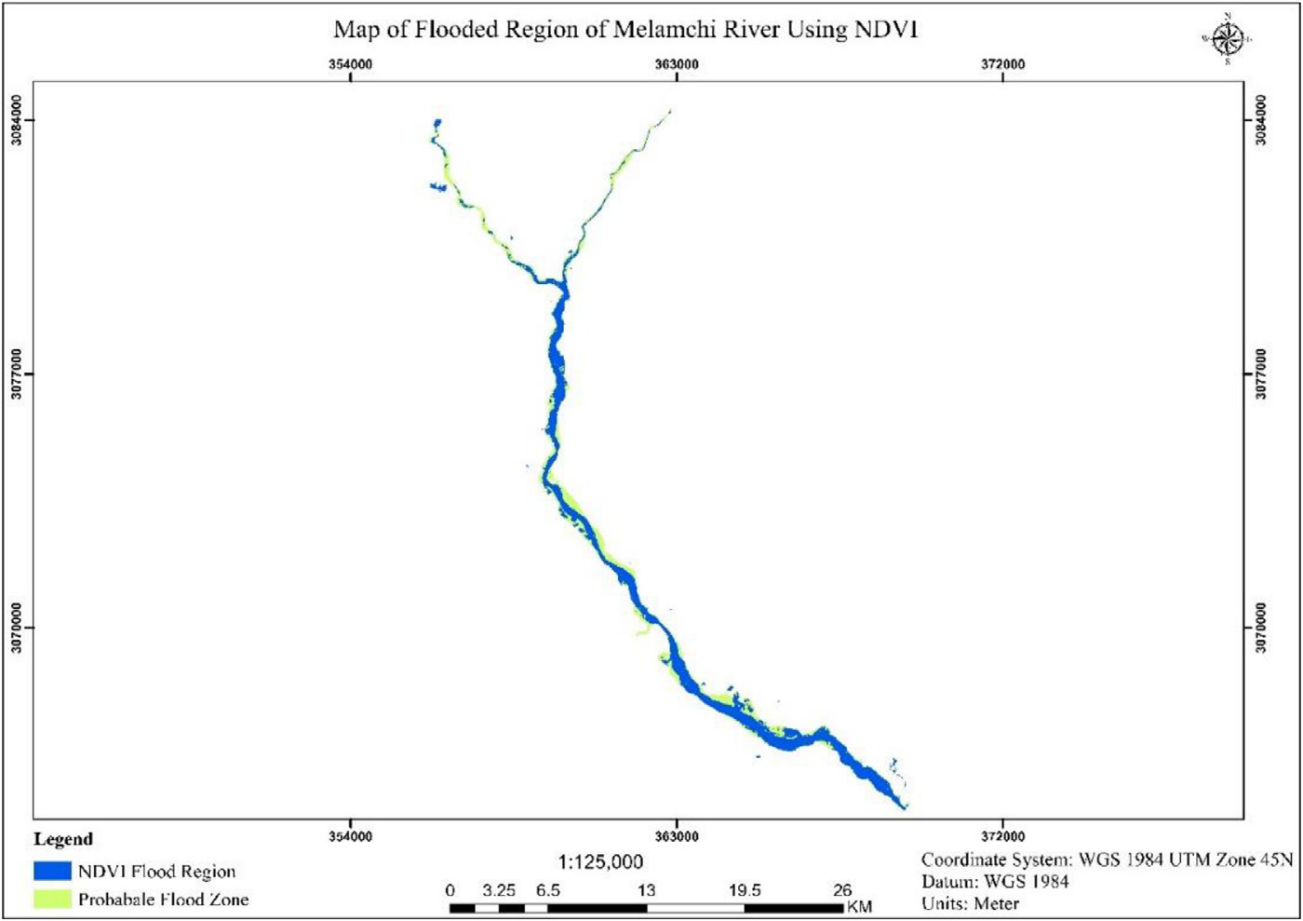
The map demonstrates how the NDVI threshold method is applied to detect flood-affected areas in the hilly terrain.

The Sentinel-2 Level-2A surface reflectance imagery was used to map annual flood extents from 2019–2023 and was processed on the Google Earth Engine platform. The analysis focused on the Hilly and Himalayan regions of Nepal (Figs. 1, 2).

To generate cloud-free composites, monsoon-season imagery (June to September) was used. Mosaic compositing was applied to reduce the influences of atmospheric noise and outliers. In cases where pixels remained cloud-affected, gap-filling was performed using the post-monsoon mosaic composite from November to February of the following year.

Cloud-affected pixels were identified using the Sentinel-2 Scene Classification Layer (SCL). Specifically, classes corresponding to cloud shadow, low, medium, high, and cirrus clouds were masked. The masked areas were then filled using cloud-free pixels from the post-monsoon images.

The Sentinel-2 satellite is equipped with the Multispectral Instrument (MSI) that captures data across the thirteen spectral bands. These spectral bands are defined by specific wavelength intervals, each designed to detect particular surface characteristics based on how different materials absorb and reflect electromagnetic energy. Among these bands, the visible range includes three bands: Blue, Green, and Red. Band 4 (Red), with a wavelength range from 640 nm to 680 nm, strongly absorbs chlorophyll, helping to distinguish vegetation [9]. The Near-infrared (NIR) Band 8, ranging from 780 nm to 900 nm, is strongly reflected by healthy vegetation [10]. Thus, these two bands are used to calculate Normalized Difference Vegetation Index (NDVI), which is useful for distinguishing between water and other non-vegetated surfaces, and plays a crucial role in flood mapping [11].

The mathematical equation to determine NDVI:

$$\text{NDVI}=\frac{NIR-Red}{NIR+Red}$$

The resulting NDVI value ranges from −1 to 1. Open water bodies typically have NDVI values below 0, whereas values between 0 and 0.2 indicate bare soil or sediment-Landen mud conditions, often associated with flood events [[12], [13], [14]]. NDVI was selected for this study because it effectively captures the mixed surface conditions typical of floods in the Himalayan region, where flood water often carries sediments, stones, and organic debris such as wood.

Thresholding was applied regionally:

Hilly region: NDVI < 0.019

Himalayan region: NDVI < 0

These thresholds were determined based on statistical analysis (mean, maximum, and minimum values) of flooded pixels across the regions. In the Himalayan region, snow masking was necessary, since NDVI values below 0 may also indicate snow. Snow-covered areas were removed using the Copernicus Global Land Cover dataset of 2021–2022, where pixels classified as permanent snow and ice were excluded. The code is available at this link.

### 4.2 Validation

The dataset was validated by comparing the area of the actual flood event, which was digitized, with the area captured using the threshold method in the major flooded region of Melamchi. The results showed that 82 % of the flooded area was correctly detected, 8 % of the area was falsely identified as flooded, and 18 % of the actual flooded area remained undetected.

**Table:** 

Band ratio	Classified (Ha)	Misclassified (Ha)	Undetected (Ha)	Digitized flooded zone (Ha)
NDVI	584	54	126	731

## Limitations

### Cloud mask accuracy

Cloud cover during the monsoon season limits the usability of Sentinel-2 optical data for flood mapping in mountainous Nepal. Although cloud masking using the Sentinel-2, Scene Classification Layer (SCL) helps to exclude cloudy pixels, misclassification of bright surfaces like snow and undetected thin clouds remains an issue. In this study, to overcome it, temporal gap-filling with post-monsoon imagery partially addresses this issue, but does not fully eliminate cloud gaps. To improve flood mapping accuracy, future work should integrate Sentinel-2 with complementary datasets such as Landsat optical imagery [15] and Sentinel SAR, which can penetrate clouds and provide all-weather coverage. Multi-sensor harmonization enhances spatial-temporal completeness and reduces uncertainty in flood risk assessment.

Spatial Resolution: The spatial resolution of Sentinel-2 imagery is 10 m, which is sufficient for detecting large-scale flooding; however, it limits the detection of small-scale or narrow flooded features such as streams, irrigation channels, or urban drainage overflow. High-resolution datasets, e.g., commercial imagery or Unmanned Aerial Vehicle (UAV) data, may be required for detailed mapping at finer scales.

Threshold Sensitivity: The NDVI threshold values used for flood detection are region-specific and may vary depending on vegetation density, soil type, sediment load, and water turbidity. These thresholds may not generalize well across different climatic zones or flood types, and calibration may be needed for application in other regions.

Cloud-Free Imagery Availability: The availability of cloud-free imagery during peak monsoon periods is limited due to persistent cloud cover. Despite the use of post-monsoon composites for gap-filling, some flood-affected areas may remain undetected, especially in regions with prolonged cloud cover.

Temporal Limitations and Seasonal Bias: The flood extent was mapped using seasonal composites, which may not capture short-duration or flash flood events accurately. Additionally, the reliance on a limited number of scenes during the monsoon season could introduce temporal bias, especially if major flood events occurred outside the compositing window.

## Ethics Statement

The authors have read and followed the ethical requirements for publication in Data in Brief and confirm that the current work does not involve human subjects, animal experiments, or any data collected from social media platforms.

## Credit Author Statement

Narayan Thapa and Sujan Nepali were responsible for the conceptualization and methodology of the study. Data curation, script development, and formal analysis were carried out by Narayan Thapa. The investigation was conducted by Sujan Nepali, Narayan Thapa, and Suman Sanjel. Sujan Nepali also contributed to data visualization. The original draft was written by Narayan Thapa, Sujan Nepali, and Raman Shrestha, while the review and editing process was collaboratively performed by Sujan Nepali, Narayan Thapa, Raman Shrestha, and Suman Sanjel. Reviewer comments were addressed by Narayan Thapa and Sujan Nepali.

## Data Availability

zenodoTime series flood mapping using the Copernicus dataset in Google Earth Engine of Mountainous Region (Original data). zenodoTime series flood mapping using the Copernicus dataset in Google Earth Engine of Mountainous Region (Original data).

## References

[1] (1982). NLC, Natural Calamity (Relief) Act. http://www.lawcommission.gov.np.

[2] Liu Y., Zhou K., Xia Q. (2018). A MaxEnt model for mineral prospectivity mapping. Natural Resources Res..

[3] Morrow B.H. (1999). Identifying and mapping community vulnerability. Disasters.

[4] Rogers David, Tsirkunov Vladimir, Cost and benefits of early warning systems, 2011. https://documents1.worldbank.org/curated/en/609951468330279598/pdf/693580ESW0P1230aster0Risk0Reduction.pdf (accessed September 13, 2024).

[5] Government of Nepal Ministry of Home Affairs, Disaster Risk Reduction and Management Act, 2074 And Disaster Risk Reduction and Management Rules, 2076 (2019), 2019.

[6] Shakti P.C. (2009). Proceedings of the International Urban Water Conference.

[7] Danegulu A., Karki S., Bhattarai P.K., Pandey V.P. (2024). Characterizing urban flooding in the Kathmandu Valley, Nepal: the influence of urbanization and river encroachment. Natural Hazards.

[8] C.-M. Chen, J. Hollingsworth, M. Clark, D. Zekkos, D. Chamlagain, S. Bista, A. Siwakoti, A.J. West, The 2021 Melamchi Flood: a massive erosional cascade in the Himalayan Mountains of central Nepal, (2023). 10.21203/RS.3.RS-2766739/V1.

[9] Holben B., Justice C. (1981). An examination of spectral band ratioing to reduce the topographic effect on remotely sensed data. Int. J. Remote Sens..

[10] J. Grabska, C.W. Huck, Near-Infrared Spectroscopy in Bio-Applications, (2020). 10.3390/molecules25122948 PMC7357077 32604876

[11] ESA, SENTINEL-2 User Handbook, European Space Agency, 2015. 10.1021/ie51400a018.

[12] Atefi M.R., Miura H. (2022). Detection of flash flood inundated areas using relative difference in NDVI from Sentinel-2 images: a case study of the August 2020 event in Charikar, Afghanistan. Remote Sensing 2022.

[13] Elkadiri R., Sultan M., Youssef A.M., Chase R., Bulkhi A.B., Al-Katheeri M.M. (2014). A remote sensing-based approach for debris-flow susceptibility assessment using artificial neural networks and logistic regression modeling. Ieeexplore.Ieee.Org.

[14] Sidi Almouctar M.A., Wu Y., Kumar A., Zhao F., Mambu K.J., Sadek M. (2021). Spatiotemporal analysis of vegetation cover changes around surface water based on NDVI: a case study in Korama basin. Southern Zinder, Niger, Appl Water Sci.

[15] Claverie M., Ju J., Masek J.G., Dungan J.L., Vermote E.F., Roger J.C., Skakun S.V., Justice C. (2018). The Harmonized Landsat and Sentinel-2 surface reflectance data set. Remote Sens. Environ..

